# Disequilibrium and complexity across scales: a patch-dynamics framework for organizational ecology

**DOI:** 10.1057/s41599-023-01730-x

**Published:** 2023-05-06

**Authors:** Jin Xu, Joep Cornelissen

**Affiliations:** 1 School of Economics and Management, South China Normal University, Guangzhou, China; 2 Rotterdam School of Management, Erasmus University Rotterdam, Rotterdam, The Netherlands

**Keywords:** Business and management, Economics, Business and management

## Abstract

Based on equilibrium assumptions, traditional ecological models have been widely applied in the fields of management and organization studies. While research using these models is still ongoing, studies have nonetheless struggled with ways to address multiple levels of analysis, uncertainty, and complexity in their analyses. This paper conceptualizes the dynamic co-evolution mechanisms that operate in an ecosystem across multiple organizational scales. Specifically, informed by recent advances in modelling in biology, a general ‘patch-dynamics’ framework that is theoretically and methodologically able to capture disequilibrium, uncertainty, disturbances, and changes in organizational populations or ecosystems, as complex and dynamically evolving resource environments are introduced. Simulation models are built to show the patch-dynamics framework’s functioning and test its robustness. The patch-dynamics framework and modelling methodology integrates equilibrium and disequilibrium perspectives, co-evolutions across multiple organization levels, uncertainties, and random disturbances into a single framework, opening new avenues for future research on topics in the field of management and organization studies, as well as on the mechanisms that shape ecosystems. Such a framework has the potential to help analyse the sustainability and healthiness of the business environment, and deserves more attention in future research on management and organization theory, particularly in the context of significant uncertainty and disturbances in business and management practice. Overall, the paper offers a distinct theoretical perspective and methodology for modelling population and ecosystem dynamics across different scales.

## Introduction

The equilibrium view represents a core concept in ecological research in management and organization studies. This view is based on the assumption of linearity and predictability. However, scholars in the fields of management and ecology have continuously questioned the equilibrium view as a basis for their theorising and modelling (Chanda and McKelvey, 2020). For instance, cooperative equilibrium is more difficult to be sustained in uncertain environments (Hergueux et al., 2021). Ecology scholars in the biological sciences likewise argue that the equilibrium view cannot fully explain the transient behaviour of ecological systems and fails to incorporate heterogeneity and multiple scales of analysis into a quantitative expression of stability (Wu and Loucks, 1995). However, to date, and despite these admissions in biology, extant theories and methodological tools in management and organizational research are mostly rooted in the concepts of equilibrium and linearity. As such, researchers tend to study the chaotic, interconnected, dynamic nature of their phenomena with tools that can only capture ‘average and limited variance’ (Andriani and McKelvey, 2009). Despite the growing emphasis that management scholars place on disequilibrium and uncertainty, the frameworks and methodology they use to describe and analyse these phenomena are, because of their roots in equilibrium thinking, largely inadequate.

Specifically, traditional ecological models, such as the NK model (Kauffman and Weinberger, 1989) and the Lotka–Volterra (LV) model have been widely used in the fields of management and organization studies for several decades. The NK model was proposed as an approach to studying strategic organising in the 1990s. Scholars in the fields of organization and management studies adopted this framework to examine the effects of environmental turbulence and complexity on the organizational design of multidepartment firms (Siggelkow and Rivkin, 2005) and to analyse the exploration and exploitation processes associated with problem-solving (Afuah and Tucci, 2012). The structure of decision-making has also been analysed using this modelling technique. For example, Rivkin and Siggelkow (2007) embedded patterned decision interactions, such as hierarchies, centralisation, and power-law distributions, into an NK simulation model to explore the need for broad exploration of firms based on different interaction patterns. Another research stream used the NK model to simulate processes of innovation (Almirall and Casadesus-Masanell, 2010; Ganco, 2017) and combinatorial search (Sorenson et al., 2006).

As a modelling technique, the NK framework is widely considered a flexible approach towards studying the outcomes of search behaviour and performance outcomes in the presence of complexity, as well as action and cognition in organizations and their interplay with search mechanisms and capability development (Gavetti, 2005). Similarly, LV modelling techniques have been used to capture how populations impact each other and to understand community processes and competition among and within populations (Amburgey and Rao, 1996; Wholey and Sanchez, 1991). These techniques and their corresponding equations have for example been used to explain and forecast the market penetration of competing technologies (Zenobia et al., 2009). As a modelling technique, the LV framework assumes that the density of competing populations has a negative impact on the establishment, growth, density and carrying capacity of the focal population (Ingram and Yue, 2008), offering a highly mathematical approach towards studying diffusion (Jarvi and Kortelainen, 2017).

Although both these ecological models have been widely used, their ability to account for complexity is limited. The key constructs on which the NK model focuses are ‘search’ and ‘speed’. Many scholars, however, call on research to identify further constructs for studying the complexity of the organizational environment (Siggelkow and Rivkin, 2005). For example, NK ‘fitness landscapes’, do not explicitly consider the interaction between players. The actions of a player have no effect on the position of other players in the same landscape (Almirall and Casadesus-Masanell, 2010). Another criticism raised about the NK model is that the agents are extremely naïve and that it is, therefore, doubtful whether the model is capable of capturing real-life dynamics and representing the process of innovation (Ganco, 2017). Similarly, the LV equations have been criticised for ignoring complexities such as third-party species, spatial structure, and life history (Smaldino et al., 2015).

Meanwhile, as industries and organizations are increasingly integrated in multiple directions (Xu et al., 2021), their boundaries are becoming fuzzier. Currently, there is no theoretical framework for studying adequately ‘different ecosystem-level structural configurations and a multiplicity of firms within an ecosystem’ (Ganco et al., 2020). It is, therefore, necessary for research to create both such a framework and a model methodology able to capture the characteristics and dynamics of organizations and populations on different scales—ranging from the agent level (e.g., the CEO or the organization) to the level of the population, the community, or the ecosystem—and in a dynamic way. However, to date, the aim of developing modelling techniques informed by a theory that captures the uncertainty and disequilibrium of complex dynamic environments simultaneously on different levels remains an important obstacle that organization scholars urgently need to overcome.

In this paper, we develop a general patch-dynamic theoretical framework that focuses on the mediating role of resources, rather than focal agents, as capturing interactions among different levels of analysis. We assumed that all participants in the ecosystem have the same opportunity to reach any patch in the system. A patch is a discrete habitat area that can accommodate populations or organizations. As organizations and populations obtain resources, the agents inside the patch-dynamic model will not be as naïve as in an NK model. Our simulation model considers the two most relevant levels of the organization, namely the patch level and the population level. Our findings reveal that, unlike previously used models, patch-dynamic frameworks and the related modelling methodology are able to capture the characteristics and dynamics of organizations and populations across different levels of analysis simultaneously. In that regard, our paper offers a different perspective for understanding strategic organising in management and organization research. The patch-dynamics framework we use allows us to integrate disturbance and stochasticity. Previously, management and organization research mostly focused on a single level of analysis—usually, either the firm level or the population or industry level. That approach, however, has unavoidable limitations as it does not capture interactions between different levels.

Our paper makes three primary contributions. First, we identify the underlying co-evolution mechanism across multiple organizational levels and scales. Second, we develop a patch-dynamics framework to capture uncertainty and disturbances in complex and dynamic resource environments. Third, we provide an empirical test of the theoretical framework and modelling techniques by building simulation models to demonstrate the mechanisms at work and the capabilities and potentials of the patch-dynamics framework. Overall, our study provides a new perspective and insights for understanding population and ecosystem dynamics in the context of organizations.

## Theoretical background

### The equilibrium perspective

In management and organizational research, evolution and revolutionary change have traditionally been conceptualised as a form of ‘punctuated’ equilibrium. Revolutionary change will interrupt long-term equilibrium (i.e., stability), radical and evolutionary innovation contrasts in the industry. Miller and Friesen (1984) tried to model momentum and revolution in the context of organizational adaptation. Systems in transition first undergo “a breakdown of the old equilibrium and a period of uncertainty about the future, before choosing a new basis around which to crystallize a new deep structure’ (Gersick, 1991).

Organizations, groups, and teams that achieve ‘punctuated equilibrium’ evolve through change, revolution and strategic reorientations (Gersick, 1988). Gersick (1991) introduced and compared models of punctuated equilibrium in the areas of organizational development and biological evolution, illustrating their potential application in organization studies, and proposed that systems would benefit from stability during equilibrium periods. ‘System equilibrium’ means that the push and pull from opposite directions are equal so the system is in balance. *Punctuated* equilibrium is ‘a stepped continuum of change in an organizational system, from revolutionary discontinuous change to more incremental change, reflecting the different levels of its deep structure’ (Chang et al., 2003). *Static* equilibrium indicates a system in a steady state, assuming that the system will react to imbalance action and regain equilibrium. More recently, Smith and Lewis (2011) presented a dynamic-equilibrium model of organising that shows ‘how purposeful and cyclical responses to paradox enable sustainability’ and organizational success. They designed their model to meet the requirements of studying increasingly dynamic and contradictory organizational environments. Their dynamic-equilibrium model reconceptualises organising and organizational change in complex contexts.

### The disequilibrium perspective

In ecology, the assumption that there is a ‘balance of nature’ has been historically popular. This assumption implies that ecological systems will revert to a previous state of equilibrium, despite disturbances—an assumption that underlies the paradigm and related concepts of point equilibrium and static stability in ecology. However, equilibrium is rare in nature. Heterogeneity and ‘scale multiplicity’ have proven difficult to integrate into quantitative expressions, challenging the classic equilibrium view (Wu and Loucks, 1995). To make linear approximations of the metacommunity (a set of communities that are linked by multiple potentially interacting species; Wilson, 1992), Wang and Loreau (2016) assumed that ‘the metacommunity has a stable equilibrium in the absence of environmental fluctuations’ and that populations will fluctuate around their equilibria. The authors admit that this assumption is one of the limitations of their research, as it did not allow them to explore sufficiently the complex and dynamic metacommunity.

Disequilibrium mechanisms are regarded as important means of explaining species diversity in different communities in ecology, they were originally examined in relation to dynamic frameworks for studying non-spatial populations. For example, Huston (1979) used LV competition models, which do not explicitly consider spatial processes, to illustrate disequilibrium mechanisms. Bartha et al. (1997) further clarified the necessity for considering spatial structure in research on persistence and coexistence. The hierarchical patch-dynamics paradigm, which emerged from such research, proposes a framework for examining heterogeneity on multiple scales, making it possible to integrate equilibrium and disequilibrium perspectives. Ever since the patch-dynamics model was introduced, ecologists have been developing it to answer important research questions. For instance, Amarasekare and Possingham (2001) put forward a model that considers patch-dynamics and metapopulation factors to investigate how the frequency of disturbances affects equilibrium patch occupancy.

### Challenging traditional ecological models

The doubts that have been expressed about the equilibrium view have led some scholars to challenge the NK model and Lotka–Volterra model, two of the most widely used ecology models in the fields of management and organization. Management and organization scholars have exploited the ability of LV models to capture dynamic phenomena in order to examine organizational decision-making processes and interactions (Carroll, 1981). Joshi et al. (2009) used a discrete two-stage representation of an LV model to describe the interactions between two markets. Jayanthi et al. (2009) applied an LV model to capture dynamic resource allocation and decision-making processes involving a firm and its manufacturing operations. In this stream of research, researchers derive phase plots, which express the relationships between two or more dynamic variables over time, by solving the set of LV differential equations with respect to equilibrium predictions.

In the field of ecology, scholars found that the empirical data certain studies presented did not agree with the predictions of LV models (Hall, 1988). One explanation for this disagreement is that the LV equations and their extensions are based on the assumption that communities are closed and isolated and populations interact directly with each other. For instance, the linear additive structure of the LV model did not allow (Bartha et al., 1997) to demonstrate the ‘true coexistence’ (Roxburgh et al., 2004) of species in competitive communities. The classic LV model used in population dynamics cannot capture ecological processes that involve interactions between and within populations on a large scale (Leibold et al., 2004). Scholars in the field of community ecology realised that the assumptions on which the LV model is based are inadequate for studying the distribution and interaction of populations on different spatial scales. This is because the linear structure that yields equilibrium solutions in the LV model constrains its capability to capture uncertainty and dynamics on different levels.

The NK model, in turn, has been frequently used in the fields of organization science and strategy management since the 1990s. Levinthal and Warglien (1999) used the NK model to emphasise that one of the most important tasks for managers is to choose a ‘landscape’ to which stakeholders can adapt. Generally, scholars used the parameters *N* (i.e., the number of influence factors or choices) and *K* (i.e., the number of interactions between various choices or factors) to jointly construct so-called fitness landscapes (Baumann et al., 2019). The NK model enabled scholars to envisage how organizations search for and find profitable solutions in landscapes (Felin et al., 2014). NK models perform well in examining the dynamics of selective evolutionary processes (Kauffman, 1993), showing how organizations change and adapt (Levinthal, 1997), as well as processes of organizational learning and innovation (Rivkin, 2000, 2001), decision-making (Ethiraj and Levinthal, 2009), and strategy positioning (Csaszar and Levinthal, 2016; Gavetti et al., 2017).

Scholars simulate resource-searching behaviour (Billinger et al., 2014; Giannoccaro et al., 2020) and problem-solving in complex systems (Baumann and Siggelkow, 2013) as local searches in a fitness landscape. Mihm et al. (2003) presented a model of a design group that creates non-tractable multipeaked fitness landscapes. Each member makes distributed decisions inside a component, while the performance of the system evolves according to how the various components interact. Rahmandad (2019) constructed and tested different aspects of performance landscapes. Mohsen and Nasiry (2020) applied the NK landscape search model to study how misalignments affect the performance of product development teams and simulated search processes involving a number of interactive teams on interdependent landscapes. Ganco et al. (2020) extended the standard NKC framework (Ethiraj and Levinthal, 2004) to obtain the NKCf (NKC input–output flows) model, therefore proving that upstream and downstream firms can benefit from each other in a complex system.

One limitation of the landscape metaphor and the related computational methods is that they require complete information on a given environment. The NK model is built on the assumption that all possible interactions in a landscape are listed and categorised (Sommer and Loch, 2004), and organizations access the value of alternatives with full certainty (Baumann and Siggelkow, 2013). However, in reality, changes occur randomly, landscapes cannot be predefined and possible interactions are unpredictable. Also, the concepts of ‘search’ and ‘landscape’ could not explain the origins of innovation and heterogeneity toward randomness and opportunities in the fields of entrepreneurship and strategy (Felin et al., 2014). Moreover, NK models focus on a single landscape scale, which means that they cannot capture the complexity and uncertainty of dynamic environments. Studies that use NK models may therefore ‘often underestimate the importance of connectivity for species occurrence and persistence’ (Hodgson et al., 2009). To relax the exogenous nature of the landscape, a new class of models is required (Ganco and Hoetker, 2009). To some extent, using the concept of a fixed or given landscape, as a ‘context’, to analyse the evolution of organisms in the economic sphere is problematic.

Over the last few decades, research on ecological processes in biology and ecology has been shifting towards the ‘micro’ level of analysis. To construct an ecosystem scale, researchers use multiple habitat patches and include interacting living creatures with similar characteristics and structures(Birgé et al., 2016). Some ecologists argue that such research needs to reconsider its basic assumptions and focus on how different landscape levels interact and call on researchers to take into account both the patch and the landscape scale (Meyer et al., 2007; Moustakas et al., 2009).

In the fields of management and organization studies, the potential of these changes in ecological models has yet to be fully explored and implemented. Ecologists underline the importance of patch-dynamics frameworks for observing effects on multiple scales and identifying how different levels interact. The hierarchical nature of patch-dynamics frameworks overcomes the problems associated with the assumptions of linearity and equilibrium underlying the LV model and can help visualise interactions in patchy and hierarchical landscapes (Poole, 2002). To date, only a few studies on ecosystems have developed ‘a truly multi-species approach in eco-evolutionary dynamic analysis’ (De Meester et al., 2019), so the patch-dynamics perspective needs to be explored further. In the fields of biology and ecology, the pros and cons of using the metaphor and methodology of the patch-dynamics approach are becoming increasingly popular. However, the advantages and disadvantages of the patch-dynamics perspective remain unclear in management studies. We address this gap by developing a patch-dynamics framework and simulation model to remedy some of the inevitable shortcomings of the equilibrium view and traditional ecological models in the management field.

### The patch-dynamics framework as a tool

A ‘patch’ is a discrete area of habitat capable of holding populations or communities (Leibold et al., 2004). Watt (1947) was the first to examine the patch-dynamics of ecological systems while Hutchinson (1953) was the first to describe the interactions within and among disequilibrium communities occupying different patches and to explain the coexistence of populations in patchy environments. Since then, the paradigm and frameworks of patch-dynamics have been researched in relation to different types of ecosystems (Wu and Levin, 1997). In terms of scale, a patch can range from a single cell to a vast landscape (Thorp et al., 2006). The patch-dynamics framework deconstructs landscapes into patches that represent ‘any division or heterogeneity in resources’ (Antolin et al., 1991) and are ‘relatively homogeneous unit[s] recognised in a mosaic at any scale’ (Forman, 1995). The patch is an important determinant of population and community dynamics (Ims et al., 2004). As a patch-dynamic system is composed of patches in different states of transition, the concept is key to understanding how different populations coexist within an ecosystem. For example, multi-patch frameworks can be used to analyse mutualistic interactions between plants and pollinators (Amarasekare and Possingham, 2001) and are considered ‘the most robust way’ to evaluate the life-history strategies of tropical trees (López-Hoffman et al., 2007).

Patch-dynamics models can simulate the size of resident populations in interconnected habitat patches (Fahrig and Gray, 1985). These models perform so well that the patch-dynamic perspective has become one of the dominant views in the fields of population ecology, community ecology and landscape ecology. The patch-dynamic view makes both theoretical and empirical generalisations possible because it captures the fundamental characteristics of an ecological system (Wu and Loucks, 1995). Patch-dynamics models are ‘conceptually simple’ and easier to use for testing data (Hugueny and Cornell, 2000). Keymer et al. (2000) emphasised the value of patch dynamics and developed a mean-field model to estimate metapopulation persistence. Xu et al. (2006) developed a model that ‘combines the features of the spatially realistic Levins model with dynamic changes in patch quality’. Gravel et al. (2010) expanded a structured landscape model to a multi-species patch dynamics model to study a nutrient–plant–detritus ecosystem, which allowed them to integrate the dynamic feedback between processes on the ecosystem and the population level.

Since Hutchinson (1953) introduced the patch-dynamics framework in community ecology, the framework has evolved into two perspectives: the landscape ecology perspective and the metacommunity perspective. The landscape ecology perspective focuses on the impact of spatial patterns on ecological processes across different scales of space and time, while the metacommunity perspective emphasises the importance of disturbances in maintaining disequilibrium communities (Winemiller et al., 2010). The metacommunity concept helps us understand increasingly complex mechanisms that work across multiple scales and provides insights that we cannot obtain through approaches that simply focus on local communities (Leibold et al., 2004). There are two levels of community integration in a metacommunity: a local level and a regional level. The *local* level is where population interactions within a local community take place. Typically, studies use the LV model to explain such interactions. A habitat containing multiple localities and able to support a metacommunity is described as a *region* and interactions between different communities occur on the regional level.

Given that non-local factors can affect the environment, it is necessary to understand population dynamics within ecosystems on a broad scale (Arroyo-Esquivel and Hastings, 2020). Mouquet and Loreau (2003) proposed the concept of ‘meta-ecosystem’ as a natural extension of the metapopulation and metacommunity concepts, and argued that this concept has the potential to integrate the perspectives of community, ecosystem, and landscape ecology. Gravel et al. (2010) presented a meta-ecosystem framework to explain the process of environmental change and its impact on individuals within patches, populations, and communities.

The patch dynamics framework is a conceptual approach that recognises spatial patterning for various ecological criteria (including individuals, populations, communities, ecosystems, and landscapes). Patches may vary with regard to environmental conditions. Patch-dynamics models can capture spatial effects and help predict environmental effects (Leibold and Loeuille, 2015). For that reason, the patch-dynamics model is regarded as a ‘useful tool in metapopulation viability analysis for comparing species persistence’ (Johansson et al., 2012). Patch-dynamics models are more flexible than traditional models when used for similar purposes and can capture better the characteristics of populations, they deserve to be further explored.

### Uncertainty and disturbance

Generally, uncertainty and disturbances have always been important topics in management and organization studies. ‘Future heterogeneity in performance is induced by a combination of present heterogeneity in some parameters plus random factors’ (Lippman and Rumelt, 2003). Unforeseeable uncertainty brings more improvement potential for maxima solutions in the true projected landscape (Sommer and Loch, 2004). In an organizational context, the diversity and number of organizations and industries that survive and recover from sudden disturbances can all be used to measure the health of such ecosystems.

Environmental disturbances lead to instability and dynamics on various scales (Wu and Loucks, 1995). Bartha et al. (1997) define a disturbance as ‘a multi-species, spatiotemporal pattern of mortality of non-competitive origin’. Because different populations have different response mechanisms to disturbances, ecosystem-level responses to natural disturbances (e.g. a disaster) can be used to measure the fitness of the ecosystem (Whitford et al., 1999). The degree of population survival, productivity, diversity, mutualistic interactions and the rate of recovery after disturbances indicate an ecosystem’s levels of resistance and resilience (Johnson, 2000). Disturbances are important in research on community diversity and are regarded as drivers of successional cycles (Meyer et al., 2007; Roxburgh et al., 2004). Disturbance and randomness contribute to the coexistence and stability of species (Buiatti and Longo, 2013). The richness and spatial distribution of species on the regional and local levels help maintain the stability of an ecosystem. Environmental homogenisation (i.e., biodiversity loss) will decrease the stability of the ecosystem (Wang and Loreau, 2016). Sudden environmental disturbances conflict with deterministic hypotheses and with the concept of equilibrium.

Importantly, patch-dynamics frameworks can show how natural disturbances produce different types of patches in natural ecosystems, they take into account disturbance and stochasticity, which are key elements in ecosystem analysis (Webb and Scanga, 2001). Disturbances cumulate to the ecosystem scale via patches (Birgé et al., 2016). Hastings and Wolin (1989) linked stochastic events associated with deterministic population growth to the patch-dynamic model. Patchy disturbances create heterogeneous structures and dynamics in a community (Ledger et al., 2008). Patches can influence the response of ecosystems to environmental disturbances and accelerate the formation of other creatures (Meloni et al., 2017).

The patch-dynamic framework is a general and useful framework that can ‘capture ecosystem dynamics spanning several scales of observation [and] allow the integration of most of the mechanisms’ (Meyer et al., 2007). The patch-dynamics framework can help organization researchers to address difficulties that they urgently need to overcome. In this paper, we apply the patch-dynamic framework to analyse interactions among landscape, patch, and population scales in the business environment. Related equations and formulas are provided in the Model specification section.

### Model specification

The standard LV equations that researchers use to analyse different species in the ecosystem are: $\frac{{{\rm {d}}N_1}}{{d_t}}$ = $r_1N_1\left( {\frac{{K_1 - N_1 - \alpha _{12}N_2}}{{k_1}}} \right)$, $\frac{{{\rm {d}}N_2}}{{d_t}}$ = $r_2N_2\left( {\frac{{K_2 - N_2 - \alpha _{21}N_1}}{{k_2}}} \right)$, where *N*_1_ is the density of population 1, *r*_1_ represents the growth rate of population 1, *K*_1_ stands for the carrying capacity of the industry environment, *α*_12_ denotes the effect of organizational population 1 on the growth of organizational population 2, and *α*_21_ denotes the reverse effect. To examine the implications of the model for organizational diversity, Hannan and Freeman (1977) extend the above equations to *M* competitors, write the model of Lotka–Volterra system as $\frac{{{\rm {d}}N_i}}{{d_t}}$ = $r_iN_i\left( {\frac{{K_i - N_i - \sum \alpha _{ij}N_j}}{{k_i}}} \right)$, *i* = 1, 2, …, *M*. Ruef (2000) also used a similar model to represent multiple competing populations with different identities in the community. Jayanthi et al. (2009) use the LV model to study the dynamics of resource allocation and the interactions between the firm and the manufacturers it employs. The authors modified the equation as follows: $\frac{{{\rm {d}}x_i\left( t \right)}}{{d_t}}$ = $\alpha _ix_i\left( t \right)\left[ {1 - \mathop {\sum }\limits_{j = 1}^2 \alpha _{ij}x_j\left( t \right)} \right],\,i = 1,\,2,$ where *x*_*i*_(*t*) denotes the trajectory of resources allocated to the firm (*i* = 1) and the manufacturer (*i* = 2) during time *t*, while *α*_*ij*_ indicates the interaction coefficient of the firm and the manufacturer. These examples show that the LV model that is widely used in management and organization studies considers the growth rate of the population, the carrying capacity of the environment, and the interaction effect of different populations in the system. What this model lacks, however, is the ability to capture the impact of environmental change, disturbances, and stochasticity.

### Model A: an extension of the LV model

Simulations are useful to develop theories regarding nonlinear phenomena or in situations when empirical data are difficult to obtain. With the focus of organization and strategy management scholars moving from equilibrium to dynamic perspectives, simulations have become a prevalent methodology for theory development (Davis et al., 2007). To test the capability and potential of the patch-dynamics framework in capturing disturbance and stochasticity, we built a simulation model based on the equations of Wang and Loreau (2016) and consider a landscape that consists of *m* patches in a stochastic environment. We assumed that each population has the same opportunities to reach any patch and used the general equation presented below to characterise the dynamics between different species in the same patch:

1 $$\begin{array}{l}\frac{{{\rm {d}}N_{al}\left( t \right)}}{{{\rm {d}}t}} = r_{al}N_{al}\left( t \right) \cdot \left( {1 - \frac{{N_{al}\left( t \right) + c_{abl} \cdot \mathop {\sum }\nolimits_{b \ne a} N_{bl}\left( t \right)}}{{K_{al}}}} \right)\\ \qquad\qquad+ \left( { - d_aN_{al}\left( t \right) + \mathop {\sum }\limits_{p \ne l} N_{ap}\left( t \right) \cdot \frac{{d_a}}{{m - 1}}} \right)\\ \qquad\qquad+\, N_{al}\left( t \right) \cdot E_{al}\,\left( t \right)\end{array}$$

Here, *N*_*al*_ (*t*) is the density of population *α* in patch *l* at time *t*, while *r*_*al*_ represents the growth rate of the population *a* in patch *l*, and *K*_*al*_ indicates the carrying capacity of population *a* in patch *l*. Additionally, *c*_*abl*_ denotes the competition coefficient of population *b* on population *a* in patch *l*, while *d*_*a*_ shows the dispersal and emigration rate of population *a*, and *N*_*bl*_ (*t*) indicates the population density of species *b* in patch *l*. Finally, *N*_*ap*_ (*t*) is the density of population *a* in patch *p*. As Eq. (1) is a general equation that characterises the dynamics of any species in any patch, *N*_*bl*_ (*t*) and *N*_*ap*_ (*t*) follows the same dynamics. Note that *E*_*al*_(*t*) shows the response of population *a* to environmental fluctuations in patch *l* and, therefore, reflects the impact of environmental change, disturbances, and stochasticity on the population. We formulate the environmental response as

2 $$E_{al}\left( t \right) = \gamma _l\left( t \right) + \delta _a\left( t \right) + \rho \cdot \gamma _l\left( t \right) \cdot \delta _a\left( t \right)$$

**Fig. 1:**
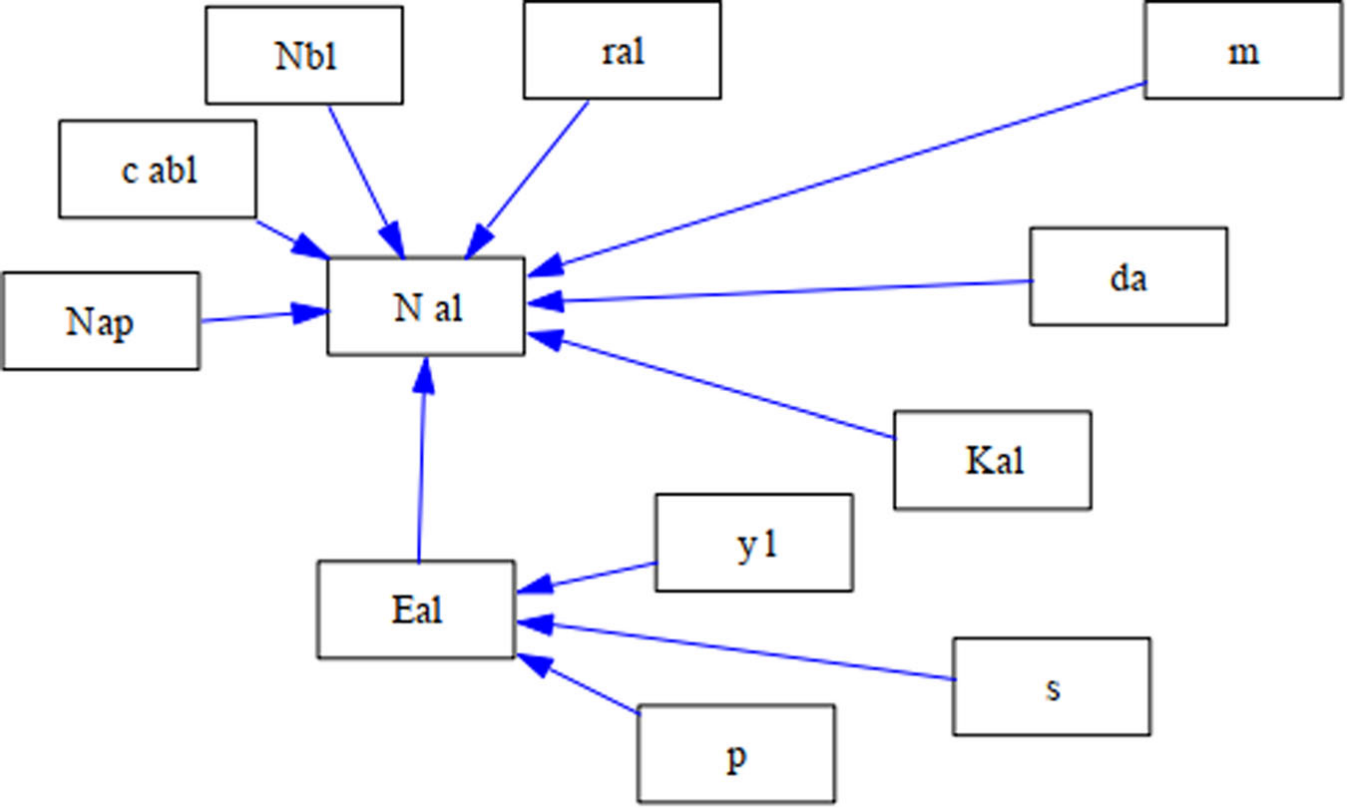
Causal loop diagram of Model A.

*γ*_*l*_(*t*) represents the patch-specific environmental responses of population *a* in patch *l* at time *t*, while *δ*_*a*_ indicates the population-specific environmental responses of population *a* in patch *l*. Additionally, $\gamma _l\left( t \right) \cdot \delta _a\left( t \right)$ represents the interaction between *γ*_*l*_ (*t*) and *δ*_*a*_ (*t*), while the strength of this interaction is determined by parameter *ρ*. Figure 1 illustrates the relationships between the variables and the calculations of model A. For more setting details, see Table 1 in Appendix A.

**Fig. 2:**
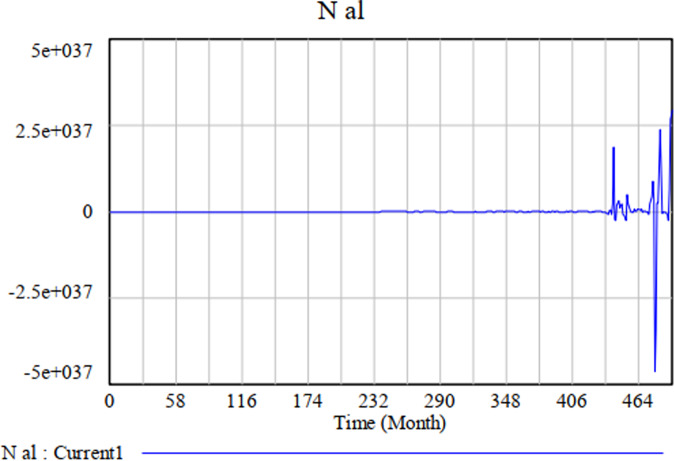
The trajectory of the density of population *a* in patch.

To test Model A, we applied the equations presented above and obtained the following simulation results: Initial Time = 0, Final Time = 492, Time Step = 1. Figure 2 shows the trajectory of the density of population *a* in patch *l*. As the figure shows, Model A can capture sudden changes in the environment.

**Fig. 3:**
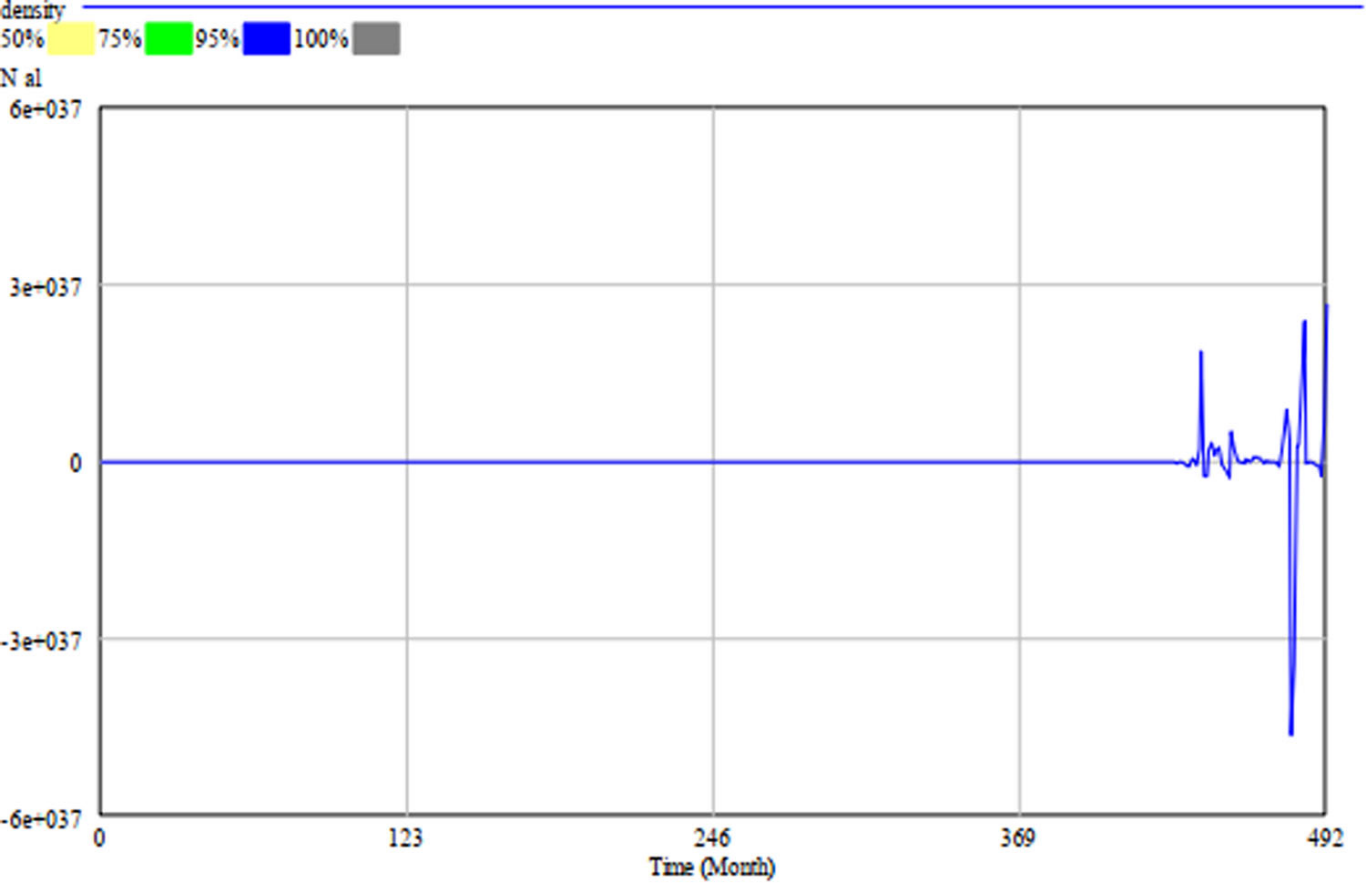
The result of the sensitivity test for *N*_*al*_.

To test the impact of uncertainty on the simulation model, we performed a multivariate sensitivity simulation of Model A in Vensim DSS. We set the number of simulations to 1000 and used 10 as Noise Seed. The simulation results remain the same when we change the minimum and maximum values of *m*, *N*_*ap*_, *N*_*bl*_ and *ρ*_*γ*_. Hekimoğlu and Barlas (2016) suggest using uniform distribution to carry out sensitivity analyses of system dynamics models. We, therefore, broadened the data range of *m* to (0, 10) and changed the value of *N*_*ap*_ from constant to a data range from 0 to 20 following the Random Uniform distribution. Figure 3 shows the results of the sensitivity test we conducted to measure the density of population *a* in patch *l* (*N*_*al*_). The diagram presents the confidence-bound regions, within which the population density occurs with 50, 75, 95, and 100 per cent probability. The narrow confidence bounds suggest that the dynamics of the population density do not change when the input values change. Therefore, we can conclude that the model is stable and robust.

### Model B: An example of the patch-dynamics framework

To illustrate the mechanisms underlying population and ecosystem dynamics based on the patch-dynamics framework, we further build Model B. Our model considers the two scales that are most relevant to organizations, i.e., the patch and population scales. To examine the mechanisms, we observe in more detail, we assume that the ecosystem’s resources are exchanged between occupied and empty patches. The model is represented by the following dynamic equations:

3 $$\frac{{{\rm {d}}N_{\rm {o}}}}{{{\rm {d}}t}} = R - {{{\mathrm{O}}}}N_{\rm {o}} - \mathop {\sum }\limits_i a_iN_{\rm {o}}Q_{ai} - L\left( {N_{\rm {o}} - N} \right)$$

*N*_o_ stands for resources in an occupied patch, *R* is the resource input from the environment, O is the resource output, *a*_*i*_ indicates the rate at which the organizations in the patch consume the resource, *Q*_*ai*_ represents the quantity of population *i* in patch *a* and *L* is the loss of the resource in the occupied patch. The resource in the empty patch *N*_e_ is given by Eq. (4):

4 $$\frac{{{\rm {d}}N_{\rm {e}}}}{{{\rm {d}}t}} = R - {{{\mathrm{O}}}}N_{\rm {e}} - L\left( {N_{\rm {e}} - N} \right)$$

5 $$\frac{{{\rm {d}}Q_{ai}}}{{{\rm {d}}t}} = a_iN_{\rm {o}}Q_{ai} - d_iQ_{ai}$$

where *d*_*i*_ stand for the death rate of population *i*. The remaining items are identical to those in Eqs. (3) and (4).

Furthermore, *N* represents the regional average; that is:

6 $$N = pN_{\rm {o}} + \left( {1 - p} \right)N_{\rm {e}}$$

Here, *p* is the spatial occupancy of patches. Occupancy is the probability a taxon occurs in the patch, offering a way to assess the spatial distributions of populations. Spatial occupancy has been widely used for population monitoring and has become a useful tool for ecologists to learn the population dynamics over time and space (Johnson et al., 2013). *N*_o_ and *N*_e_ represent resources in the occupied patch and empty patch, respectively. We use the spatial occupancy of patches to capture the dynamics of a population and calculate the spatial occupancy of a patch by:

7 $$\frac{{{\rm {d}}p_i}}{{{\rm {d}}t}} = c_ip_i\left( {1 - \mathop {\sum }\limits_{j = 1}^i p_j} \right) - mp_i - \mathop {\sum }\limits_{j = 1}^{i - 1} c_jp_jp_i$$

where *p*_*i*_ is the spatial occupancy of the specific patch *i*, *m* is the disturbance rate in the patch, and *c*_*i*_ indicates the colonisation rate; that is, the number of resources produced per patch. *c*_*i*_ is calculated by the number of resources per population produced and multiplied it by *Q*_*ai*_. Figure 3 shows the causal loop diagram of Model B. Table 2 in the Appendix provides more details on the parameter setting in the simulation of Model B.

**Fig. 4:**
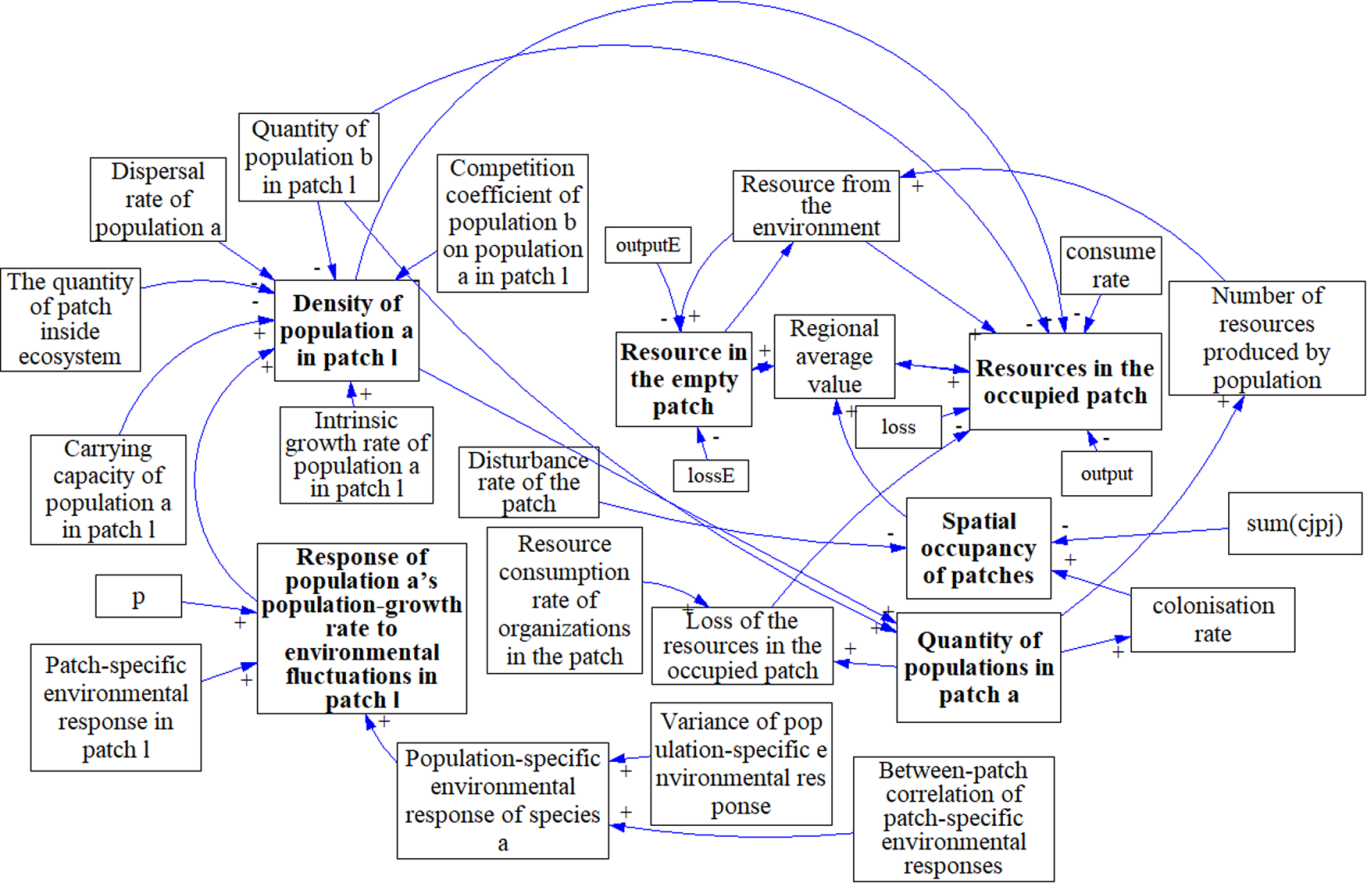
Causal loop diagram of variables in our patch-dynamic framework.

**Fig. 5:**
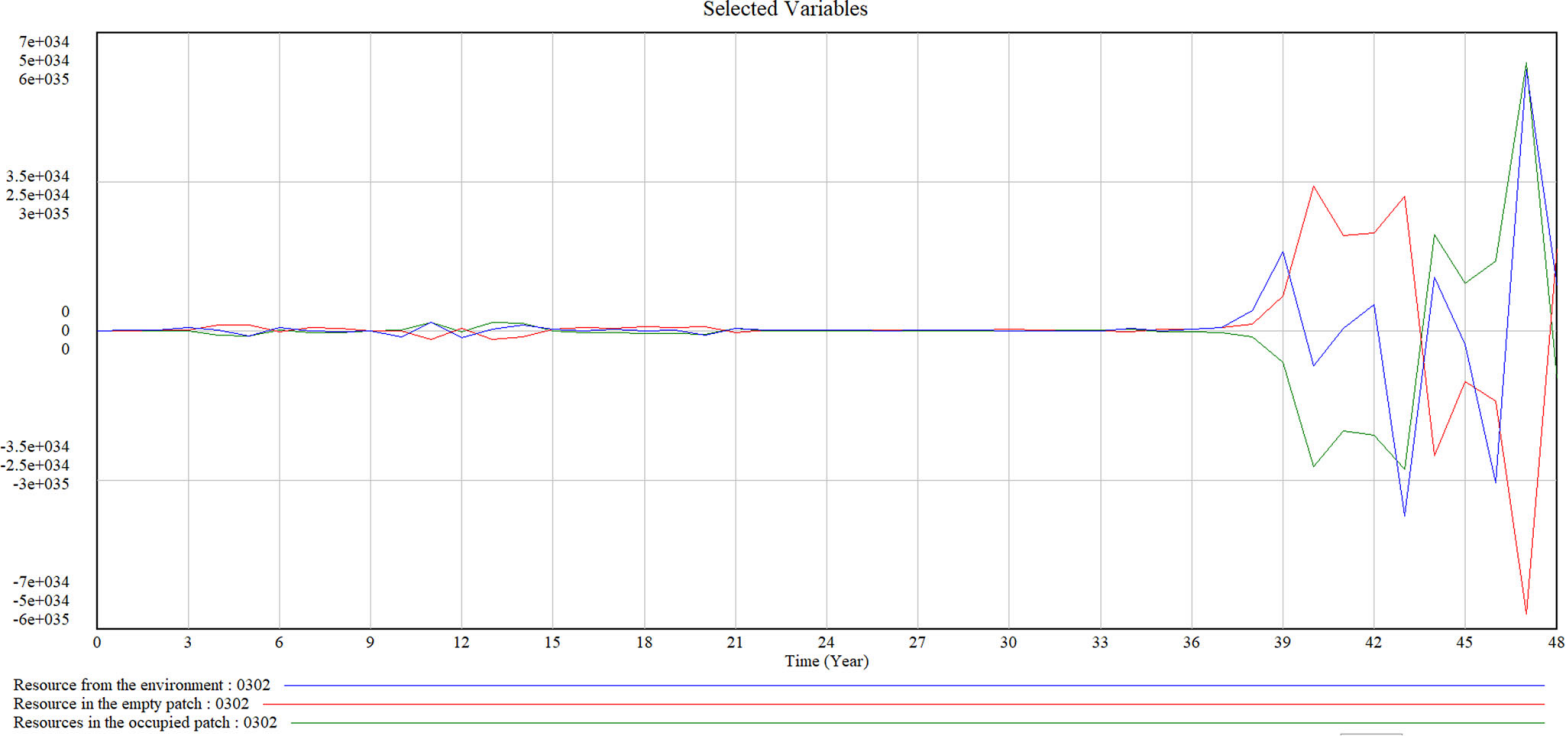
Simulation results of resources from the environment and resources in the occupied and empty patch.

Figure 4 shows the causal loop diagram of factors we considered in our patch-dynamics framework. We highlight the key variables in bold font so that how the previous models connect could be easily spotted. We set the units for a time from ‘Month’ to ‘Year’, in line with the premise that the patch-dynamics framework is more suitable to express longer time phases. In Model B, we integrate the patch scale with the population and ecosystem scales, taking these organization-relevant scales into consideration. The transaction of resources from the environment to the patch and between occupied and empty patches are captured in the model. Furthermore, we show the mechanisms between the impact of environmental disturbances on population densities inside the patch, therefore revealing the reactions of the population to random disturbances. The simulated results of resources from the environment and resources in the occupied and empty patch created by Model B are shown in Fig. 5.

**Fig. 6:**
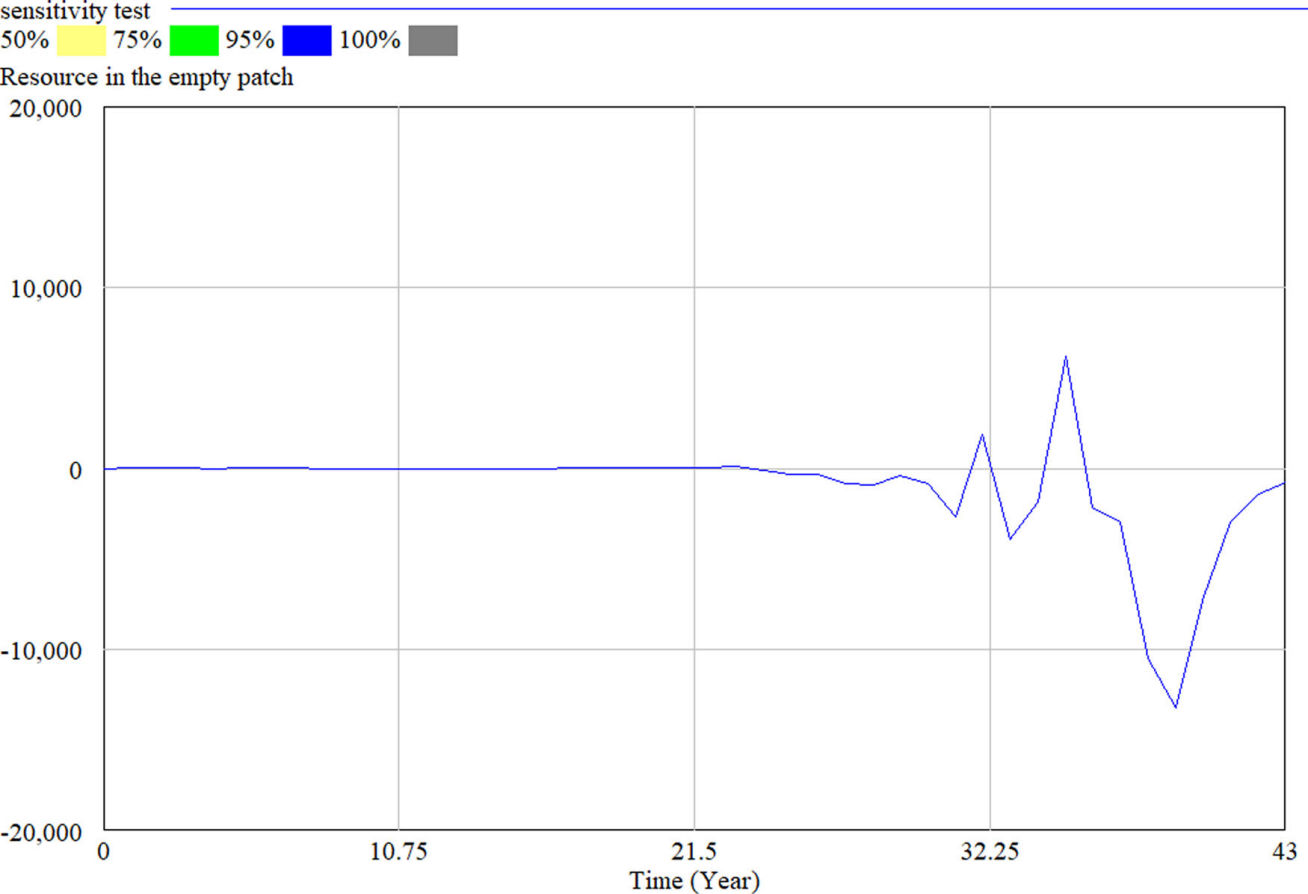
The result of the sensitivity test for resources in the occupied patch.

In the sensitivity analysis used, we apply the Monte Carlo multivariate sensitivity simulation (MVSS) method so that all parameters will change together automatically. The method works by sampling numbers from within a set range of values, so that “the distribution for each parameter specified is sampled” (Vensim User Guide, 2023). As we set the number of simulations at 2000, this process is repeated 2000 times. We further set 1234 as the noise-seed value and ran the simulation 2000 times to obtain the sensitivity graph. We broadened the data range to a maximum of 10 times, changed the resource consumption rate of organizations in the patch to Random Uniform (0, 10), the loss of the resources in the occupied patch to (0, 100), broadened the disturbance rate of the patch to (0, 10), and ran the software to create automatically random sample values for the parameters according to our specifications (Fig. 6). We see that the trajectory of resources in the occupied patch does not change significantly when we vary the input values, model B is robust.

Validating the model involves ensuring that the simulation analysis fits the context and requirements of the research topic (Troost et al., 2023). The structural validity of the simulation model is “a stringent measure” (Vensim User Guide, 2023) to enhance confidence in the simulation model. Boundary adequacy and dimensional consistency tests can furthermore be used for structural validation of the system dynamics model. A boundary adequacy test aims to test if the concepts and structures are endogenous to the model (Qudrat-Ullah and Seong, 2010), identifying the degree to which significant concepts can endogenously address the research question. “If parameters are likely to change over time, they should be endogenized” (Schwaninger and Groesser, 2018). We carefully checked all the parameters and equations of the model, confirming that the parameters shown in the above figures change over time. Schwaninger and Groesser (2018) also mentioned that the indirect version of a boundary adequacy behaviour test involves testing if the model behaviour changes significantly when the boundary was extended or reduced. The model contains concepts that are important for addressing the problem endogenously. Meanwhile, from the above sensitivity tests (Figs. 3 and 6), we could validate the boundary adequacy in this indirect way and pass the test. The dimensional consistency test is an additional powerful tool for testing the internal validity of a model (Schwaninger and Groesser, 2018). The test focuses on checking the measurement units of all the parameters of the model, aiming to make sure all of them are dimensionally consistent. As the variables and constants in each mathematical equation of the patch-dynamics model are dimensionless, it already shows dimensional consistency. As the patch dynamics model passed the boundary adequacy and dimensional consistency tests, we can also confirm the structural validation of the model.

## Results

We find some interesting results based on our patch-dynamic modelling. Firstly, the simulation shows that resources in the occupied, empty patch and in the environment are stable in the beginning (From Year 0 to Year 37 in Fig. 5). The sudden increase of resources from the environment occurs in Year 37, reaching the maximum value point at Year 39. Resource in the empty patch has a converse development trend compared with the resource in the occupied patch, which make sense as organizations and populations will consume resources when they survive in the patch. Resource in the empty patch shows a similar tendency but around one year delay as resources from the environment, also increased and last for one more year until Year 40. Meanwhile, resource in the occupied patch shows a decreasing tendency until Year 40. Although the population inside the patch needs to consume the resources offered by the ecosystem, they produce resources and contribute to the patch and environment at the same time, which explains the sudden increase of resources in the environment and resources in the occupied patch after Year 43. As resources in the environment decrease after reaching their maximum, resources in the empty patch will still grow for another 1 year (until Year 48). During this specific period, companies in the ecosystem figure out ways to survive, as they create a balance between the population quantity in the patch and the resources that they need to sustain their living. These results further prove that the patch-dynamic framework has the potential to describe the intentional actions of organizations vis-a-vis populations.

**Fig. 7:**
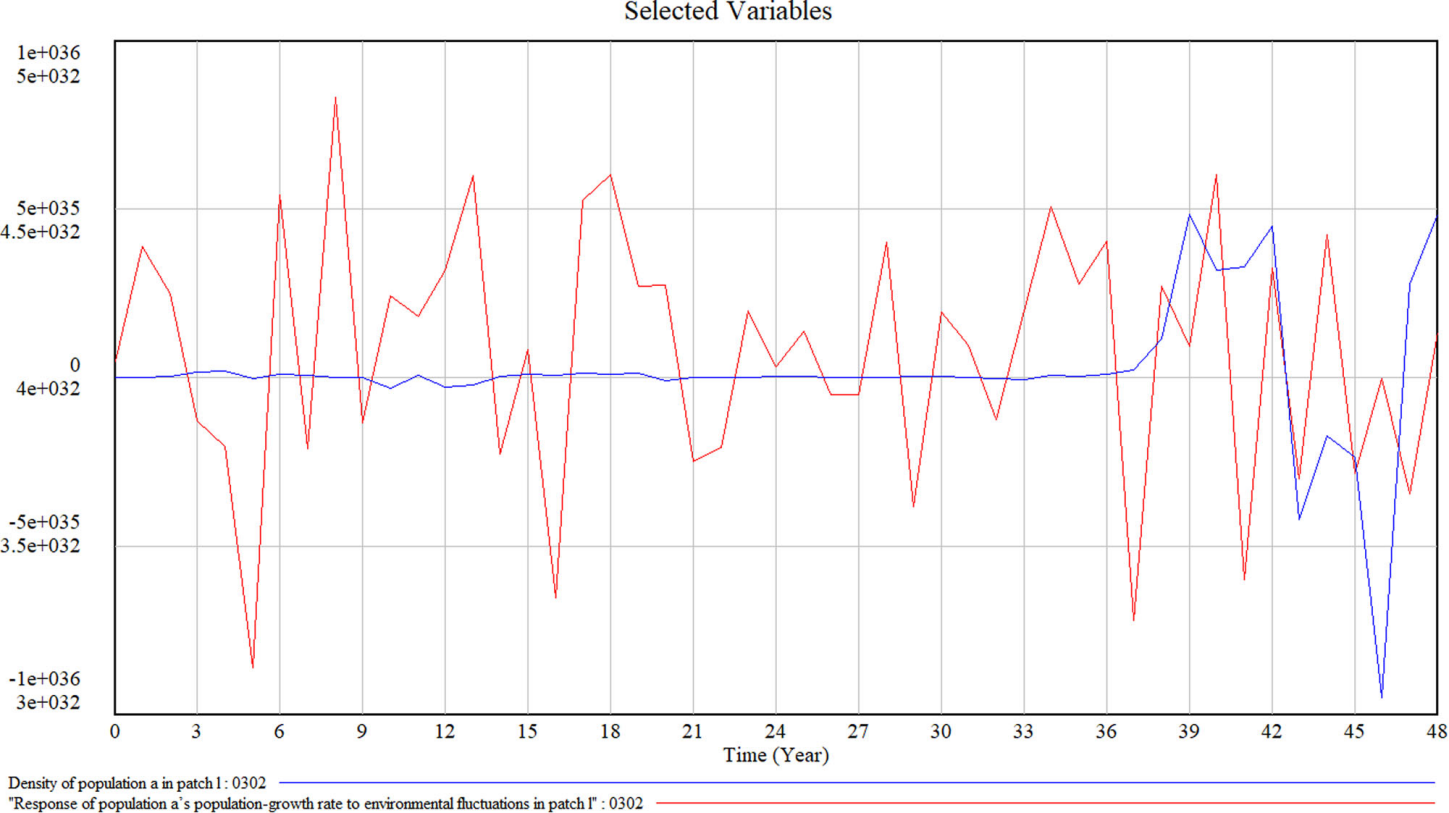
Simulation results of the response of the population’s population-growth rate to environmental fluctuations and density of population in patch l.

Figure 7 presents an overview of the population’s reaction to environmental fluctuations. As the environment changes frequently in the initial stage, the density of the population in the patch remains stable in the lower level. After around 36 years after the emergence of the population in the patch, population density starts to increase. But still fluctuates in the same direction as the environment change, although in a 2-year delay. For instance, from year 36, when the response of population a’s population-growth rate to environmental fluctuations in patch l reaches its maximum in year 37, the density of the population in the patch also reaches the highest point at year 39. The simulation results prove that the organizational population has a delay of 2 years when reacting to environmental changes, reflecting the time it needs for decision-makers of the organizations and populations to learn and realise changes in response to their environment.

**Fig. 8:**
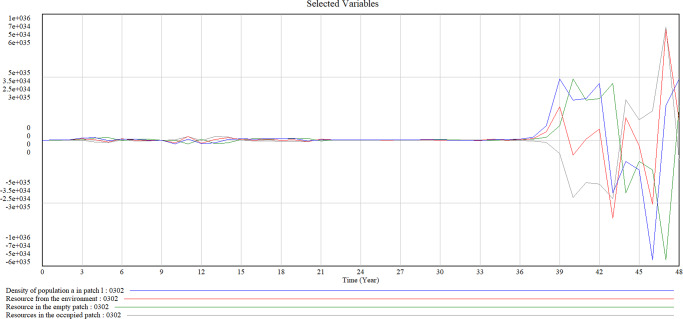
Simulation results of the density of population, resources in the empty and occupied patch.

When we further observe the density of the population in a specific patch and compare this with trends in resource evolution between the occupied patch and empty patch, we obtain Fig. 8. Figure 8 shows that the density of the population and resources in the ecosystem is stable in the early phase, but after 36 years, the population density inside the patch increases dramatically while resources from the environment and empty patches grow. When resource from the environment fluctuates, resources in the occupied patch shows almost the same trend. Developments of the population further increase the stock of resources that contribute to the empty and occupied patch. Therefore, resources in the occupied patch display higher value since Year 43 and rebound even though resources from the environment and empty patch suffer from a decrease after Year 45. This phenomenon helps the density of the population recover from the lowest point (Year 46) and represents a significant rebound. In all, our simulation proves the capability of the patch-dynamic framework in describing such complex and evolving changes over time.

## Discussion

Our patch-dynamics framework has certain implications for research on strategic search (e.g., search for resources, innovative products, or technologies) and provides macro-insights into the behavioural theory of organizations. Existing research often visualises a landscape with peaks and valleys within which innovators can explore or exploit. ‘Search’ is a core process of strategic decision-making. Many scholars focus on the structure and processes of a given landscape and the trade-offs between exploration and exploitation. Kneeland et al. (2020), for example, applied the NK framework to show the mechanisms through which individuals form mental representations of reality and how specific processes facilitate innovation and exploration in the technological landscape. More recently, Billinger et al. (2021) studied the processes through which individuals decide whether and how they behave when given a complex task. Most of these studies, however, examine search as a form of isolated decision-making, neglecting the ‘complexities of competitive interactions in search [processes]’ (Billinger et al., 2021), while, in reality, individuals, organizations, and populations can observe and learn from others. As the boundaries of organizations and industries become fuzzier (Xu et al., 2021), it is necessary for scholars to examine organizations and populations across different organization levels, instead of focusing on a single scale. Our framework captures the characteristics and dynamics of organizations and populations on diverse scales and levels. Specifically, our paper bridges landscapes, organizations, and populations and provides a more comprehensive approach to understanding strategic decision-making and search processes on different scales.

Economists are being criticised for focusing either on the equilibrium or the disequilibrium state (growth state); economic equilibrium tells only one side of the story (Sunny, 2020). The analysis by Wang et al. (2021) is based on static Nash equilibria, they argued that future research could assess their findings in a dynamic setting by exploring dynamic decision-making on platforms. Heterogeneity makes it more difficult to achieve equilibrium in an ecosystem, while plurality triggers innate paradoxical tensions and the decisions of individuals may harm the health of an ecosystem (Wareham et al., 2014). For instance, developers inside the platform ecosystem prefer to protect their intellectual property (IP) but hope other developers open their IP. Parker and Van Alstyne (2018) also found that the necessary cooperation to achieve IP and R&D spillovers in a platform ecosystem is not a pure strategy equilibrium. Conflicts between actors’ personal interests and broader benefits for the entire ecosystem become significant when the quantity and diversity of those actors increase. Complexity and heterogeneity decrease the possibility of achieving equilibrium for the current economics and platform ecosystem. Disequilibrium and disruptions contribute to innovation, strategy-as-practice and technological change literature (Farjoun, 2019). Our patch-dynamics framework integrates the equilibrium and disequilibrium perspectives and provides general equations for characterising the dynamics of organizations and populations in any patch. Our framework furthermore enriches our understanding of the dynamics and the complex interactions between and within organizations, populations, and ecosystems. Having the capability to capture characteristics and complexities across multiple organization levels inside the ecosystem, our patch-dynamics framework contributes to the literature on the dynamic evolution of organizations, populations, and ecosystems.

Another important implication of our paper’s findings is that organization theorists need to theorise and systematically study the impact of random disturbances and uncertainties on populations and ecosystems. Our research offers insights into how organizations and populations respond to environmental fluctuations and uncertainties and proposes a methodology for capturing the impact of environmental change, disturbances, and stochasticity on economic entities. Previous research on entrepreneurship and strategy has offered limited explanations for randomness and opportunities (Felin et al., 2014). The patch dynamics model uses the spatial occupancy of patches to capture the dynamics of populations and allows for varying environmental conditions. It can therefore capture immigration from nearby patches and predict both the spatial effects and environmental effects of the ecosystem on organizations and populations.

Last but not least, the importance of the platform economy and digital ecosystems has increased rapidly in recent years. Emerging digital ecosystems may generate a total of more than $60 trillion in revenue and constitute 30% of global corporate revenue by 2025. Market-leading platform enterprises and market winners tend to have the biggest share of orders and revenue, while other companies face increasing pressures to survive. The misuse of such market power may give some companies an unfair competitive advantage, constrain the diversity of the ecosystem and ultimately harm consumers, small businesses, and social welfare (Rietveld and Schilling, 2021). Parker and Van Alstyne (2018) emphasized that it is necessary to consider the impact of the merger on the multiple sides of the ecosystem. Large platform-based enterprises, such as Uber and Amazon, may destroy the resource habitat of other companies and decrease the diversity of organizations and activities inside the entire community. However, is such a ‘winner-take-all’ phenomenon sustainable for the general ecosystem and environment? How do we measure the fitness and healthiness of the entire ecosystem? The current methodology, which focuses on a single level of analysis, cannot address these questions adequately.

## Conclusion and limitations

The patch-dynamics framework and modelling methodology have the potential to address such questions, however, and to help analyse the sustainability and healthiness of the business environment. The patch-dynamic approach helps to capture how an ecosystem responds to environmental disturbances, thus improving our understanding of ecosystem fitness (Johnson, 2000). More generally, patch-dynamics frameworks help explain the fundamental mechanism of biodiversity conservation (Liao et al., 2017). Overall, the patch-dynamics view and framework deserve more attention in future research on management and organization theory, particularly in the context of the COVID-19 pandemic, which has created significant uncertainty and disturbances in business and management practice. The COVID-19 crisis has led to many changes in economic systems, global supply chains, and leadership around the world. The patch-dynamics framework integrates equilibrium and disequilibrium perspectives, co-evolutions across multiple organization levels, uncertainties, and random disturbances into a single framework, opening new avenues for future research on these topics in the field of management and organization studies, as well as on the mechanisms that shape ecosystems.

Due to data limitations, empirical research using real data is beyond the scope of this paper. Future research may engage in further behaviour pattern tests to explore how well the model mimics the behaviour of the real system modelled and thus identify whether the real system would exhibit similar sensitivity as the simulations display (Qudrat-Ullah and Seong, 2010). Such behavioural sensitivity tests may then indicate how “model-based recommendations influenced by uncertainty” change in terms of the values of their model parameters (Schwaninger and Groesser, 2018). With this general limitation noted, our research is a first attempt to show the functioning of the patch-dynamics framework with the overall aim being to offer a theoretical perspective and methodology for modelling population and ecosystem dynamics across different scales. Future research may apply data from specific industrial ecosystem settings to test the theoretical framework we proposed and inspire continuous insightful exploration in this direction.

## Supplementary information

Appendix Disequilibrium and complexity across scales: a patch-dynamics framework for organizational ecology

## References

[CR1] Afuah A, Tucci CL (2012). Crowdsourcing as a solution to distant search. Acad Manag Rev.

[CR2] Almirall E, Casadesus-Masanell R (2010). Open versus closed innovation: a model of discovery and divergence. Acad Manag Rev.

[CR3] Amarasekare P, Possingham H (2001). Patch dynamics and metapopulation theory: the case of successional species. J Theor Biol.

[CR4] Amburgey TL, Rao H (1996). Organizational ecology: past, present, and future directions. Acad Manag J.

[CR5] Andriani P, McKelvey B (2009). From Gaussian to Paretian thinking: causes and implications of power laws in organizations. Organ Sci.

[CR6] Antolin MF, Addicott JF, Antolin MF, Addicott JF (1991). Colonization, among shoot movement, and local population neighborhoods of two Aphid species. Oikos.

[CR7] Arroyo-Esquivel J, Hastings A (2020) Spatial dynamics and spread of ecosystem engineers: two patch analysis. Bull Math Biol 82(12). 10.1007/s11538-020-00833-9 10.1007/s11538-020-00833-9 33211197

[CR8] Bartha S, Czárán T, Scheuring I (1997). Spatiotemporal scales of non-equilibrium community dynamics: a methodological challenge. N Z J Ecol.

[CR9] Baumann O, Schmidt J, Stieglitz N (2019). Effective search in rugged performance landscapes: a review and outlook. J Manag.

[CR10] Baumann O, Siggelkow N (2013). Dealing with complexity: integrated vs. chunky search processes. Organ Sci.

[CR11] Billinger S, Srikanth K, Stieglitz N, Schumacher TR (2021). Exploration and exploitation in complex search tasks: how feedback influences whether and where human agents search. Strateg Manag J.

[CR12] Billinger S, Stieglitz N, Schumacher TR (2014). Search on rugged landscapes: an experimental study. Organ Sci.

[CR13] Birgé HE, Allen CR, Garmestani AS, Pope KL (2016). Adaptive management for ecosystem services. J Environ Manag.

[CR14] Buiatti M, Longo G (2013). Randomness and multilevel interactions in biology. Theory Biosci.

[CR15] Carroll GR (1981). Dynamics of organizational expansion in national systems of education. Am Sociol Rev.

[CR16] Chanda SS, McKelvey B (2020). Back to the basics: reconciling the continuum and orthogonal conceptions of exploration and exploitation. Comput Math Organ Theory.

[CR17] Chang A, Bordia P, Duck J (2003). Punctuated equilibrium and linear progression: toward a new understanding of group development. Acad Manag J.

[CR18] Csaszar FA, Levinthal DA (2016). Mental representation and the discovery of new strategies. Strateg Manag J.

[CR19] Davis JP, Eisenhardt KM, Bingham CB (2007). Developing theory through simulation methods. Acad Manag Rev.

[CR20] De Meester L, Brans KI, Govaert L, Souffreau C, Mukherjee S, Vanvelk H, Korzeniowski K, Kilsdonk L, Decaestecker E, Stoks R, Urban MC (2019). Analysing eco-evolutionary dynamics—the challenging complexity of the real world. Funct Ecol.

[CR21] Ethiraj SK, Levinthal D (2004). Bounded rationality and the search for organizational architecture: an evolutionary perspective on the design of organizations and their evolvability. Adm Sci Q.

[CR22] Ethiraj SK, Levinthal D (2009). Hoping for A to Z while rewarding only a: complex organizations and multiple goals. Organ Sci.

[CR23] Fahrig L, Gray M (1985). Habitat patch connectivity and population survival. Ecology.

[CR24] Farjoun M (2019). Strategy and dialectics: rejuvenating a long-standing relationship. Strateg Organ.

[CR25] Felin T, Kauffman S, Koppl R, Longo G (2014). Economic opportunity and evolution: beyond landscapes and bounded rationality. Strateg Entrep J.

[CR26] Forman, RTT (1995) Land mosaics: the ecology of landscapes and regions. Cambridge University Press, New York

[CR27] Ganco M (2017). NK model as a representation of innovative search. Res Policy.

[CR28] Ganco M, Hoetker G (2009) NK modeling methodology in the strategy literature: bounded search on a rugged landscape. In: Research methodology in strategy and management. Vol. 5, pp. 237–268, Emerald Group Publishing Limited

[CR29] Ganco M, Kapoor R, Lee GK (2020). From rugged landscapes to rugged ecosystems: structure of interdependencies and firms’ innovative search. Acad Manag Rev.

[CR30] Gavetti G (2005). Cognition and hierarchy: rethinking the microfoundations of capabilities’ development. Organ Sci.

[CR31] Gavetti G, Helfat CE, Marengo L (2017). Searching, shaping, and the quest for superior performance. Strategy Sci.

[CR32] Gersick CJG (1988). Time and transition in work teams: toward a new model of group development. Acad Manag J.

[CR33] Gersick CTG (1991). Revolutionary change theories: a multilevel exploration of the punctuated equilibrium paradigm. Acad Manag Rev.

[CR34] Giannoccaro I, Galesic M, Massari GF, Barkoczi D, Carbone G (2020). Search behavior of individuals working in teams: a behavioral study on complex landscapes. J Bus Res.

[CR35] Gravel D, Mouquet N, Loreau M, Guichard F (2010). Patch dynamics, persistence, and species coexistence in metaecosystems. Am Nat.

[CR36] Hall CAS (1988). An assessment of several of the historically most influential theoretical models used in ecology and of the data provided in their support. Ecol Model.

[CR37] Hannan MT, Freeman J (1977). The population ecology of organizations. Am J Sociol.

[CR38] Hastings A, Wolin CL (1989). Within-patch dynamics in a metapopulation. Ecology.

[CR39] Hekimoğlu M, Barlas Y (2016). Sensitivity analysis for models with multiple behavior modes: a method based on behavior pattern measures. Syst Dyn Rev.

[CR40] Hergueux J, Henry E, Benkler Y, Algan Y (2021) Social exchange and the reciprocity roller coaster: evidence from the life and death of virtual teams. Organization Science, Articles in Advance, pp. 1–19

[CR41] Hodgson JA, Moilanen A, Thomas CD (2009). Metapopulation responses to patch connectivity and quality are masked by successional habitat dynamics. Ecology.

[CR42] Hugueny B, Cornell HV (2000). Predicting the relationship between local and regional species richness from a patch occupancy dynamics model. J Anim Ecol.

[CR43] Huston M (1979). A general hypothesis of species diversity. Am Nat.

[CR44] Hutchinson GE (1953). The concept of pattern in ecology. Proc Acad Nat Sci Phila.

[CR45] Ims RA, Leinaas HP, Coulson S (2004). Spatial and temporal variation in patch occupancy and population density in a model system of an arctic *Collembola* species assemblage. Oikos.

[CR46] Ingram P, Yue LQ (2008). Structure, affect and identity as bases of organizational competition and cooperation. Acad Manag Ann.

[CR47] Jarvi K, Kortelainen S (2017). Taking stock of empirical research on business ecosystems: a literature review. Int J Bus Syst Res.

[CR48] Jayanthi S, Roth AV, Kristal MM, Venu LCR (2009). Strategic resource dynamics of manufacturing firms. Manag Sci.

[CR49] Johansson V, Ranius T, Snall T (2012). Epiphyte metapopulation dynamics are explained by species traits, connectivity, and patch dynamics. Ecology.

[CR50] Johnson DS, Conn PB, Hooten MB, Ray JC, Pond BA (2013). Spatial occupancy models for large data sets. Ecology.

[CR51] Johnson KH (2000). Trophic-dynamic considerations in relating species diversity to ecosystem resilience. Biol Rev.

[CR52] Joshi YV, Reibstein DJ, Zhang ZJ (2009). Optimal entry timing in markets with social influence. Manag Sci.

[CR53] Kauffman SA (1993) The origins of order: self-organization and selection in evolution. Issue Oxford University Press

[CR54] Kauffman SA, Weinberger ED (1989). The NK model of rugged fitness landscapes and its application to maturation of the immune response. J Theor Biol.

[CR55] Keymer JE, Marquet PA, Velasco-Hernandez JX, Levin SA (2000). Extinction thresholds and metapopulation persistence in dynamic landscapes. Am Nat.

[CR56] Kneeland MK, Schilling MA, Aharonson BS (2020). Exploring uncharted territory: knowledge search processes in the origination of outlier innovation. Organ Sci.

[CR57] Ledger ME, Harris RML, Armitage PD, Milner AM (2008). Disturbance frequency influences patch dynamics in stream benthic algal communities. Oecologia.

[CR58] Leibold MA, Holyoak M, Mouquet N, Amarasekare P, Chase JM, Hoopes MF, Holt RD, Shurin JB, Law R, Tilman D, Loreau M, Gonzalez A (2004). The metacommunity concept: a framework for multi-scale community ecology. Ecol Lett.

[CR59] Leibold MA, Loeuille N (2015). Species sorting and patch dynamics in harlequin metacommunities affect the relative importance of environment and space. Ecology.

[CR60] Levinthal DA (1997). Adaptation on rugged landscapes. Manag Sci.

[CR61] Levinthal DA, Warglien M (1999). Landscape design: designing for local action in complex worlds. Organ Sci.

[CR62] Liao J, Bearup D, Wang Y, Nijs I, Bonte D, Li Y, Brose U, Wang S, Blasius B (2017). Robustness of metacommunities with omnivory to habitat destruction: disentangling patch fragmentation from patch loss. Ecology.

[CR63] Lippman SA, Rumelt RP (2003). A bargaining perspective on resource advantage. Strateg Manag J.

[CR64] López-Hoffman L, Ackerly DD, Anten NPR, Denoyer JL, Martinez-Ramos M (2007). Gap-dependence in mangrove life-history strategies: a consideration of the entire life cycle and patch dynamics. J Ecol.

[CR65] Meloni F, Granzotti CRF, Bautista S, Martinez AS (2017) Scale dependence and patch size distribution: clarifying patch patterns in Mediterranean drylands. Ecosphere 8(2). 10.1002/ecs2.1690

[CR66] Meyer KM, Wiegand K, Ward D, MOUSTAKAS A (2007). The rhythm of savanna patch dynamics. J Ecol.

[CR67] Mihm J, Loch C, Huchzermeier A (2003). Problem-solving oscillations in complex engineering projects. Manag Sci.

[CR68] Miller D, Friesen P (1984) Organizations: a quantum view. Prentice-Hall, Englewood Cliffs, NJ

[CR69] Mohsen JS, Nasiry J (2020). Organizational structure, subsystem interaction pattern, and misalignments in complex NPD projects. Prod Oper Manag.

[CR70] Mouquet N, Loreau M (2003). Community patterns in source–sink metacommunities. Am Nat.

[CR71] Moustakas A, Sakkos K, Wiegand K, Ward D, Meyer KM, Eisinger D (2009). Are savannas patch-dynamic systems? A landscape model. Ecol Model.

[CR72] Parker G, Van Alstyne M (2018). Innovation, openness, and platform control. Manag Sci.

[CR73] Poole GC (2002). Fluvial landscape ecology: addressing uniqueness within the river discontinuum. Freshw Biol.

[CR74] Qudrat-Ullah H, Seong BS (2010). How to do structural validity of a system dynamics type simulation model: the case of an energy policy model. Energy Policy.

[CR75] Rahmandad H (2019). Interdependence, complementarity, and ruggedness of performance landscapes. Strategy Sci.

[CR76] Rietveld J, Schilling MA (2021). Platform competition: a systematic and interdisciplinary review of the literature. J Manag.

[CR77] Rivkin JW (2000). Imitation of complex strategies. Manag Sci.

[CR78] Rivkin JW (2001). Reproducing knowledge: replication without imitation at moderate complexity. Organ Sci.

[CR79] Rivkin JW, Siggelkow N (2007). Patterned interactions in complex systems: implications for exploration. Manag Sci.

[CR80] Roxburgh SH, Shea K, Wilson JB (2004). The intermediate disturbance hypothesis: patch dynamics and mechanisms of species coexistence. Ecology.

[CR81] Ruef M (2000). The emergence of organizational forms: a community ecology approach. Am J Sociol.

[CR82] Schwaninger M, Groesser S (2018) System dynamics modeling: validation for quality assurance. Encycl Complex Syst Sci 1–20. 10.1007/978-3-642-27737-5_540-4

[CR83] Siggelkow N, Rivkin JW (2005). Speed and search: designing organizations for turbulence and complexity. Organ Sci.

[CR84] Smaldino PE, Calanchini J, Pickett CL (2015). Theory development with agent-based models. Organ Psychol Rev.

[CR85] Smith & Lewis (2011) Toward a theory of paradox: a dynamic equilibrium model of organizing. Acad Manag Rev 36(2):381–403

[CR86] Sommer SC, Loch CH (2004). Selectionism and learning in projects with complexity and unforeseeable uncertainty. Manag Sci.

[CR88] Sorenson O, Rivkin JW, Fleming L (2006). Complexity, networks and knowledge flow. Res Policy.

[CR89] Sunny SA (2020) “Nature cannot be fooled”: a dual-equilibrium simulation of climate change. Organ Environ 1–15. 10.1177/1086026620937461

[CR90] Thorp JH, Thoms MC, Delong MD (2006). The riverine ecosystem synthesis: biocomplexity in river networks across space and time. River Res Appl.

[CR106] Troost C (2023). How to keep it adequate: A protocol for ensuring validity in agent-based simulation. Environ Model Softw.

[CR105] Vensim User Guide (2023) https://www.vensim.com/documentation/users_guide.html

[CR91] Wang L, Rabinovich E, Richards TJ (2021). Scalability in platforms for local groceries: an examination of indirect network economies. Prod Oper Manag.

[CR92] Wang S, Loreau M (2016). Biodiversity and ecosystem stability across scales in metacommunities. Ecol Lett.

[CR93] Wareham J, Fox PB, Giner JLC (2014). Technology ecosystem governance. Organ Sci.

[CR94] Watt AS (1947). Pattern and process in the plant community. J Ecol.

[CR95] Webb SL, Scanga SE (2001). Windstorm disturbance without patch dynamics: twelve years of change in a Minnesota forest. Ecology.

[CR96] Whitford WG, Rapport DJ, DeSoyza AG (1999). Using resistance and resilience measurements for ‘fitness’ tests in ecosystem health. J Environ Manag.

[CR97] Wholey DR, Sanchez SM (1991). The effects of regulatory tools on organizational populations. Acad Manag Rev.

[CR98] Wilson DS (1992). Complex interactions in metacommunities, with implications for biodiversity and higher levels of selection. Ecology.

[CR99] Winemiller KO, Flecker AS, Hoeinghaus DJ (2010). Patch dynamics and environmental heterogeneity in lotic ecosystems. J N Am Benthol Soc.

[CR100] Wu J, Levin SA (1997). A patch-based spatial modeling approach: conceptual framework and simulation scheme. Ecol Model.

[CR101] Wu J, Loucks OL (1995). From balance of nature to hierarchical patch dynamics: a paradigm shift in ecology. Q Rev Biol.

[CR102] Xu D, Feng Z, Allen LJS, Swihart RK (2006). A spatially structured metapopulation model with patch dynamics. J Theor Biol.

[CR103] Xu J, Peng B, Cornelissen J (2021). Modelling the network economy: a population ecology perspective on network dynamics. Technovation.

[CR104] Zenobia B, Weber C, Daim T (2009). Artificial markets: a review and assessment of a new venue for innovation research. Technovation.

