# Supplementary material for: Disequilibrium and complexity across scales: a patch-dynamics framework for organizational ecology
Source: Humanit Soc Sci Commun. 2023 May 6;10(1):211. doi: 10.1057/s41599-023-01730-x (PMC10163862; doi:10.1057/s41599-023-01730-x)
Supplement: Supplementary file 1 — Appendix Disequilibrium and complexity across scales: a patch-dynamics framework for organizational ecology [file 41599_2023_1730_MOESM1_ESM.pdf]

Appendix A

Table 1 Parameter-setting in the simulation of Model A

Parameter	Definition	Setting
m	Number of patches within ecosystem	m = 2
r_{al}	Intrinsic growth rate of population a in patch l	r_{al}~$runif[-2,2]$
K_{al}	Carrying capacity of population a in patch l	K_{al}~$runif[-2,2]$
c_{abl}	Competition coefficient of population b on population a in patch l	c_{abl}~$runif[0.05,1.25]$
d_a	Dispersal rate of population a	d_a~$runif[0,1]$, d_a= 0 means that this species is a specialist while d_a=1 indicates generalist
E_{al}	Response of population a's population-growth rate to environmental fluctuations in patch l	As in Equation (1.2)
γ_l	Patch-specific environmental response in patch l	γ_l~$rnorm(0, {\sigma_\gamma}^2)$, $cor(\gamma_l, \gamma_k) = \rho_\gamma$
δ_a	Population-specific environmental response of species a	δ_a~$rnorm(0, {\sigma_\delta}^2)$, $cor(\delta_a, \delta_b) = \rho_\delta$
${\sigma_\gamma}^2$	Variance of patch-specific environmental response	${\sigma_\gamma}^2 = 0.04$
${\sigma_\delta}^2$	Variance of population-specific environmental response	${\sigma_\delta}^2 = 0.04$
ρ_γ	Between-patch correlation of patch-specific environmental responses	$\rho_\gamma = -0.4$

Table 2 Parameter-setting in the simulation of Model B

Symbols	Definition	Range
Variables:		
N_o	Resources in the occupied patch	
N_e	Resource in the empty patch	
Q_{ai}	Quantity of population i in patch a	
N	Regional average value	
Parameters:		
R	Resource inputs from the environment	(0, 10)
O	Resource output	(0, 10)
a_i	Resource consumption rate of organizations in the patch	(0, 1)
L	Loss of the resources in the occupied patch	(0, 10)
d_i	Death rate of population i	(0, 1)
p	Spatial occupancy of patches	(0, 1)
m	Disturbance rate of the patch	(0.1, 0.9)
c_i	Number of resources produced per population	(0.1, 10)
